# Identification of key features required for efficient S-acylation and plasma membrane targeting of sprouty-2

**DOI:** 10.1242/jcs.249664

**Published:** 2020-11-05

**Authors:** Carolina Locatelli, Kimon Lemonidis, Christine Salaun, Nicholas C. O. Tomkinson, Luke H. Chamberlain

**Affiliations:** 1 Strathclyde Institute of Pharmacy and Biomedical Sciences, University of Strathclyde, 161 Cathedral Street, Glasgow G4 0RE, UK; 2 Institute of Molecular Cell and Systems Biology, College of Medical Veterinary and Life Sciences, University of Glasgow, Glasgow G12 8QQ, UK; 3 WestCHEM, Department of Pure and Applied Chemistry, University of Strathclyde, 295 Cathedral Street, Glasgow G1 1XL, UK

**Keywords:** Sprouty-2, Post-translational modification, PTM, S-acylation, ZDHHC enzymes, Palmitoylation

## Abstract

Sprouty-2 is an important regulator of growth factor signalling and a tumour suppressor protein. The defining feature of this protein is a cysteine-rich domain (CRD) that contains twenty-six cysteine residues and is modified by S-acylation. In this study, we show that the CRD of sprouty-2 is differentially modified by S-acyltransferase enzymes. The high specificity/low activity zDHHC17 enzyme mediated restricted S-acylation of sprouty-2, and cysteine-265 and -268 were identified as key targets of this enzyme. In contrast, the low specificity/high activity zDHHC3 and zDHHC7 enzymes mediated more expansive modification of the sprouty-2 CRD. Nevertheless, S-acylation by all enzymes enhanced sprouty-2 expression, suggesting that S-acylation stabilises this protein. In addition, we identified two charged residues (aspartate-214 and lysine-223), present on opposite faces of a predicted α-helix in the CRD, which are essential for S-acylation of sprouty-2. Interestingly, mutations that perturbed S-acylation also led to a loss of plasma membrane localisation of sprouty-2 in PC12 cells. This study provides insight into the mechanisms and outcomes of sprouty-2 S-acylation, and highlights distinct patterns of S-acylation mediated by different classes of zDHHC enzymes.

## Abstract (precis)

**Highlighted Article:** S-acylation of the tumour suppressor protein sprouty-2 requires specific cysteine and charged residues in the cysteine-rich domain, and regulates plasma membrane targeting.

## INTRODUCTION

A myriad of cell surface receptors allows cells to sense and respond to environmental cues and mediate intercellular communication. Receptor tyrosine kinases (RTKs) are integral to cell growth and division promoted by a range of different growth factors via the activation of intracellular signalling cascades, including RAS-ERK, PI3K-AKT, JAK/STAT and PLCγ pathways. However, as inappropriate activation of growth factor signalling can stimulate excessive cell growth and tumorigenesis, these signalling pathways must be tightly regulated. Sprouty proteins are negative regulators of RAS/ERK signalling via a number of mechanisms (Kawazoe and Taniguchi, 2019; Mason et al., 2006; Masoumi-Moghaddam et al., 2014), and the importance of these proteins as tumour suppressors is highlighted by their reduced expression in a range of malignancies. For example, sprouty-2 expression is downregulated in renal cell carcinoma, hepatocellular carcinoma, epithelial ovarian cancer, non-small-cell lung carcinoma, chronic lymphocytic leukaemia, gastric cancer, breast cancer and prostate cancer (Kawazoe and Taniguchi, 2019). Consistent with its function as a tumour suppressor, low expression of sprouty-2 also correlates with poorer prognosis in some of these malignancies (Kawazoe and Taniguchi, 2019; Masoumi-Moghaddam et al., 2014). Interestingly, there is also evidence that sprouty-2 has oncogenic activity, and its upregulation is associated with poor prognosis in glioblastoma patients (Kawazoe and Taniguchi, 2019; Park et al., 2018). Furthermore, the role of sprouty-2 in colorectal cancer is still controversial with some findings reporting protein upregulation (Holgren et al., 2010) and others downregulation (Feng et al., 2011; Kawazoe and Taniguchi, 2019).

Sprouty proteins were first discovered in *Drosophila melanogaster* as inhibitors of FGF signalling during development of the tracheal system (Hacohen et al., 1998). Indeed, this protein was shown to antagonize both FGF and EGF signalling, thus defining a class of general negative regulators of RTKs (Kramer et al., 1999). A striking feature of sprouty proteins is the C-terminal cysteine-rich domain (CRD) (Hacohen et al., 1998; Kramer et al., 1999). This defining feature led to the identification of four mammalian sprouty isoforms, which share the CRD and display additional strong homology with *Drosophila* Sprouty in a short stretch of amino acids that include a highly conserved tyrosine residue (tyrosine-55 in human sprouty-2) (De Maximy et al., 1999; Hacohen et al., 1998). Of all the mammalian isoforms, sprouty-2 is the most highly conserved across species and also displays the highest similarity with the *Drosophila* protein (Hacohen et al., 1998). Several studies have established mammalian sprouty proteins as inhibitors of FGF, VEGF, PDGF, BDNF, GDNF and NGF signalling (Masoumi-Moghaddam et al., 2014). Although mammalian sprouty proteins are thought to largely function as antagonists of RTK signalling, several reports suggest that sprouty proteins might exert positive modulation of EGF receptor signalling (Kim and Bar-Sagi, 2004; Wong et al., 2002).

Several protein interactions of sprouty proteins have been identified, including their homo- and hetero-oligomerization (Chen et al., 2013). Sprouty-2 is recognised and phosphorylated by several protein kinases, including Src-like kinases, which modify tyrosine-55; this phosphorylation is crucial for both sprouty-2 activity and its ubiquitylation by c-Cbl (Wai Fong et al., 2003) and subsequent degradation (Mason et al., 2004). In addition, mitogen-activated protein kinase interacting kinase 2 (Mnk2; also known as MKNK2) phosphorylates sites including serine-112 and -121 (Edwin et al., 2010), and this is thought to further regulate the level of phosphorylation at tyrosine-55 (DaSilva et al., 2006). These phosphorylation events regulate certain interactions of sprouty-2; for example, modification of tyrosine-55 is thought to generate a binding site for Grb2, leading to sequestration of this adaptor protein, thereby blocking activation of Ras and downstream ERK signalling (Hanafusa et al., 2002). Grb2 binding may also require a cryptic proline-rich site in the C-terminus of sprouty-2 that only becomes accessible following PP2A-mediated dephosphorylation of sites including serine-112 (Lao et al., 2006, 2007). Other identified binding partners of sprouty-2 include testicular protein kinase 1 (Tesk1), which may regulate Grb2 interaction (Chandramouli et al., 2008), and caveolin-1 and phosphatidylinositol (4,5)-bisphosphate (PIP2), which may be involved in plasma membrane association in response to growth factor signalling (Impagnatiello et al., 2001; Lim et al., 2002).

The CRD of sprouty proteins is required for certain protein interactions and it also plays a central role in regulating the intracellular localisation of sprouty proteins. Specifically, sprouty-2 translocates to the plasma membrane in response to growth factor signalling and this behaviour is recapitulated by the isolated CRD (residues 178–282) (Lim et al., 2000, 2002). The role of the CRD in membrane targeting may relate to its reported interaction with caveolin-1 and PIP2 (Impagnatiello et al., 2001; Lim et al., 2002). However, it has been reported that the CRD is also modified by S-acylation (Impagnatiello et al., 2001). S-acylation (also known as palmitoylation), is a process whereby fatty acids are attached onto cysteine residues. This modification is known to play an important role in membrane targeting, and could therefore contribute to the plasma membrane association of sprouty-2, or its association with various intracellular compartments. Such compartments include microtubules, vimentin filaments, and early, late and recycling endosomes (Hausott et al., 2019; Kim et al., 2007; Lim et al., 2000).

S-acylation is a widespread reversible post-translational modification (PTM) (Chamberlain and Shipston, 2015), which has a range of effects on modified proteins, including mediating stable membrane binding of soluble proteins or soluble loops of transmembrane proteins, regulating protein sorting and modulating protein stability (Blaskovic et al., 2013; Salaun et al., 2010). S-acylation is important in many cellular processes and pathways, such as modulation of signalling pathways (Rocks et al., 2010), and regulation of synaptic activity and plasticity (Matt et al., 2019). Most S-acylated proteins characterised to-date are modified on a single or a small number of cysteine residues (Chamberlain and Shipston, 2015). The CRD of sprouty-2 is particularly striking as it contains 26 cysteine residues, and it is not clear which of these cysteine residues undergo S-acylation.

In mammalian cells, S-acylation is mediated by a family of 23 S-acyltransferase ‘zDHHC’ enzymes. All zDHHC enzymes share a similar topology of several transmembrane domains and a highly conserved Asp-His-His-Cys (DHHC)–cysteine-rich domain (CRD) of 51 amino acids. The DHHC–CRD is the catalytic domain of these enzymes and is positioned at the cytosolic face of intracellular membranes, which is where the zDHHC enzymes interact with their substrates (Gottlieb and Linder, 2017; Greaves and Chamberlain, 2011; Rana et al., 2018a,b). S-acylation is a two-step process with the first step being autoacylation of the enzyme, and the second step being the transfer of the acyl chain from the enzyme to the substrate (Jennings and Linder, 2012; Mitchell et al., 2010). Within cells, zDHHC enzymes mainly localise to the Golgi and endoplasmic reticulum, with a few isoforms found at the plasma membrane or at recycling endosomes (Greaves and Chamberlain, 2011; Ohno et al., 2006). At the Golgi, where most S-acylation events are thought to take place, certain zDHHC enzymes have been classified as low-selectivity/high-activity or high-selectivity/low-activity (Lemonidis et al., 2014). Low-selectivity/high-activity isoforms include zDHHC7 and zDHHC3, which are active against a plethora of proteins and do not appear to recognise specific features of their substrates to mediate S-acylation (Chamberlain and Shipston, 2015; Lemonidis et al., 2014). The ability of these enzymes to S-acylate proteins most likely depends on other factors such as colocalisation and proximity of reactive cysteine residues in the substrate to the DHHC active site (Lemonidis et al., 2017a). High-selectivity/low-activity enzymes include zDHHC17 and zDHHC13, which require specific recognition of their substrate proteins for successful S-acylation (Lemonidis et al., 2014). The key substrate-recognition site of zDHHC17 and zDHHC13 is their N-terminal ankyrin-repeat (ANK) domain, which recognises a conserved (VIAP)(VIT)XXQP (X, any amino acid) sequence, also known as the zDABM (zDHHC ANK binding motif) in their substrates (Lemonidis et al., 2015). This zDABM occurs within disordered regions of substrates and mutation of the highly conserved proline residue in this sequence, or deletion of the ANK domain of zDHHC17, perturbs interaction and S-acylation (Greaves et al., 2009b; Lemonidis et al., 2014).

Although sprouty-2 is known to be S-acylated, there is essentially nothing else known about this PTM. Key outstanding questions include which zDHHC enzymes mediate sprouty-2 S-acylation, which cysteine residues in the CRD are modified, whether other regions of the CRD are important for S-acylation, and how S-acylation affects intracellular targeting. Here, we present the first detailed analysis of sprouty-2 S-acylation.

## RESULTS

### S-acylation of sprouty-2 is mediated by both high- and low-specificity zDHHC enzymes, and leads to increased expression levels of the protein

**Fig. 1.**
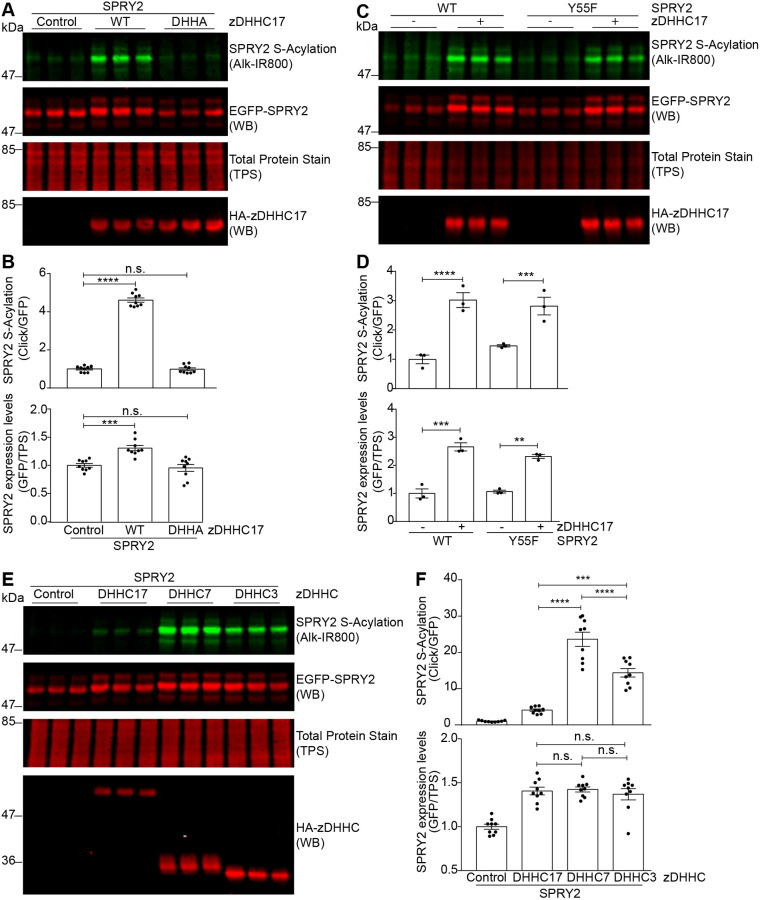
**Effects of zDHHC enzymes on the S-acylation and expression of sprouty-2 WT and its tyrosine-55 mutant.** HEK293 T cells were transfected with plasmid encoding EGFP-tagged sprouty-2 together with empty pEFBOS-HA (control in figure), HA-tagged zDHHC17 WT or zDHHA17 (A,B), EGFP-tagged sprouty-2 WT or Y55F, together with pEFBOS-HA (indicated as ‘–’ in figure), or plasmid encoding HA-tagged zDHHC17 WT (indicated as ‘+’ in figure) (C,D), or EGFP-tagged sprouty-2 together with pEFBOS-HA (control in figure), or plasmids encoding HA-tagged zDHHC17, zDHHC7, or zDHHC3 (E,F). Cells were incubated with 100 μM palmitic acid azide for 4 h and labelled proteins reacted with alkyne IRdye-800 using click chemistry. Before performing immunoblotting, membranes were incubated with a total protein stain (TPS) and the signal was detected at 700 nm. S-acylation was revealed at 800 nm; GFP and HA signals were detected at 700 nm. (A,C,E) Representative image showing sprouty-2 S-acylation (top), sprouty-2 expression levels (second row) and total protein stain (TPS, third row) detected on the same immunoblot. HA (bottom) was revealed for the same samples on a different immunoblot. The positions of the molecular weight mass are shown on the left. (B,D,F) Graphs showing sprouty-2 S-acylation and expression levels after normalisation. Results are mean±s.e.m.; each replicate is shown with filled circles. (B) Data are pooled from three independent experiments. Samples were compared with control (*n*=9). (D) Data shown is from one of two independent experiments. Samples co-expressing zDHHC17 were compared with the corresponding samples without zDHHC17 (*n*=3). (F) Data are pooled from three independent experiments (*n*=9). ****P<0.0001; ***P<0.001; n.s., not significant (one-way ANOVA).

The CRD in sprouty-2, which contains 26 cysteine residues, is modified by S-acylation (Impagnatiello et al., 2001). Our recent work showed that sprouty-2 contains a consensus recognition site for the S-acylation enzyme zDHHC17 (Lemonidis et al., 2017b), and these two proteins have also been reported to form a stable complex in cells (Huttlin et al., 2017). To examine whether zDHHC17 is able to S-acylate sprouty-2, the two proteins were co-expressed in HEK293 T cells (mouse zDHHC17 had an N-terminal 3xHA tag, whereas mouse sprouty-2 had an N-terminal EGFP tag). Cells were then incubated with C16:0-azide and labelled proteins reacted with an alkyne-modified infrared 800 (Alk-IR800) dye for detection by a LICOR infrared scanner. Fig. 1A,B shows that the S-acylation of sprouty-2 was increased when zDHHC17 was co-expressed. Furthermore, the increase in S-acylation was accompanied by a marked enhancement in the expression levels of the main sprouty-2 (i.e. fast-migrating/lower) immunoreactive band (Fig. 1A,B). To test whether the increase in sprouty-2 expression seen when it is co-expressed with zDHHC17 is dependent on S-acylation, sprouty-2 was also co-expressed with a catalytically dead version of zDHHC17 in which the cysteine of the DHHC motif (C467) was mutated to alanine. This mutant ‘zDHHA17’ retains the principal binding site for sprouty-2 (i.e. the ankyrin repeat domain). As shown in Fig. 1A,B, in the presence of this inactive zDHHA17 mutant, S-acylation of sprouty-2 was not increased above that of the negative control (i.e. empty vector pEFBOS-HA). Notably, sprouty-2 expression was also not increased by this catalytically dead mutant of zDHHC17, suggesting that the observed enhancement in sprouty-2 levels is mediated by S-acylation per se rather than binding of sprouty-2 to zDHHC17 or other effects of protein co-expression.

Previous work has shown that phosphorylation of the highly conserved tyrosine-55 (Y55) in sprouty-2 is linked to protein stability via a c-Cbl-dependent pathway. Phosphorylation at this residue has been reported to enhance the binding of c-Cbl, triggering the subsequent poly-ubiquitylation and proteasomal degradation of sprouty-2 (Kim and Bar-Sagi, 2004; Mason et al., 2006; Wai Fong et al., 2003). To investigate whether the increased expression of sprouty-2 following S-acylation by zDHHC17 is linked to this pathway, we blocked Y55 phosphorylation by utilising Y55A and Y55F mutants (Kim and Bar-Sagi, 2004; Sasaki et al., 2001; Wai Fong et al., 2003). As shown in Fig. 1C,D and Fig. S1, both sprouty-2 Y55 mutants were used as substrates by zDHHC17, and showed similar S-acylation levels to wild-type (WT) sprouty-2, suggesting that the phosphorylation state of this residue is not important for zDHHC17-mediated S-acylation. Furthermore, Y55A and Y55F expression levels were also increased to a similar level to WT sprouty-2 when zDHHC17 was co-expressed. These data suggest that the observed S-acylation-dependent enhancement in sprouty-2 levels occurs independently of the phosphorylation status of tyrosine-55.

Certain Golgi-localised zDHHC enzymes have been classified as either high-selectivity/low-activity or low-selectivity/high-activity. zDHHC17 is a high-selectivity/low-activity zDHHC enzyme that requires direct interaction with its substrates for effective S-acylation (Lemonidis et al., 2014). However, this enzyme co-localises at the Golgi with more promiscuous zDHHC enzymes, including the low-specificity/high-activity zDHHC3 and zDHHC7 (Lemonidis et al., 2014; Ohno et al., 2006). To investigate whether these more promiscuous enzymes can also modify sprouty-2, and to what extent, sprouty-2 was co-expressed with zDHHC17, zDHHC7 or zDHHC3 in HEK293 T cells (as for zDHHC17, both zDHHC7 and zDHHC3 had an N-terminal 3xHA tag). Fig. 1E,F shows that expression of either zDHHC7 or zDHHC3 resulted in a substantial increase in sprouty-2 S-acylation, far greater than that seen with zDHHC17. Despite this substantially different level of S-acylation, sprouty-2 expression was similarly increased by all three zDHHC enzymes. This latter observation, that zDHHC3, zDHHC7 and zDHHC17 enhance sprouty-2 expression to the same extent, implies that the increased S-acylation signal associated with zDHHC3 or zDHHC7 reflects the modification of a larger number of cysteine residues in the CRD by these enzymes (rather than the modification of more sprouty-2 molecules). It further suggests that S-acylation of a smaller number of cysteine residues by zDHHC17, is sufficient to promote sprouty-2 stabilisation.

### S-acylation of sprouty-2 by zDHHC17 requires cysteine residues 265 and 268, which are located in a hydrophobic patch

**Fig. 2.**
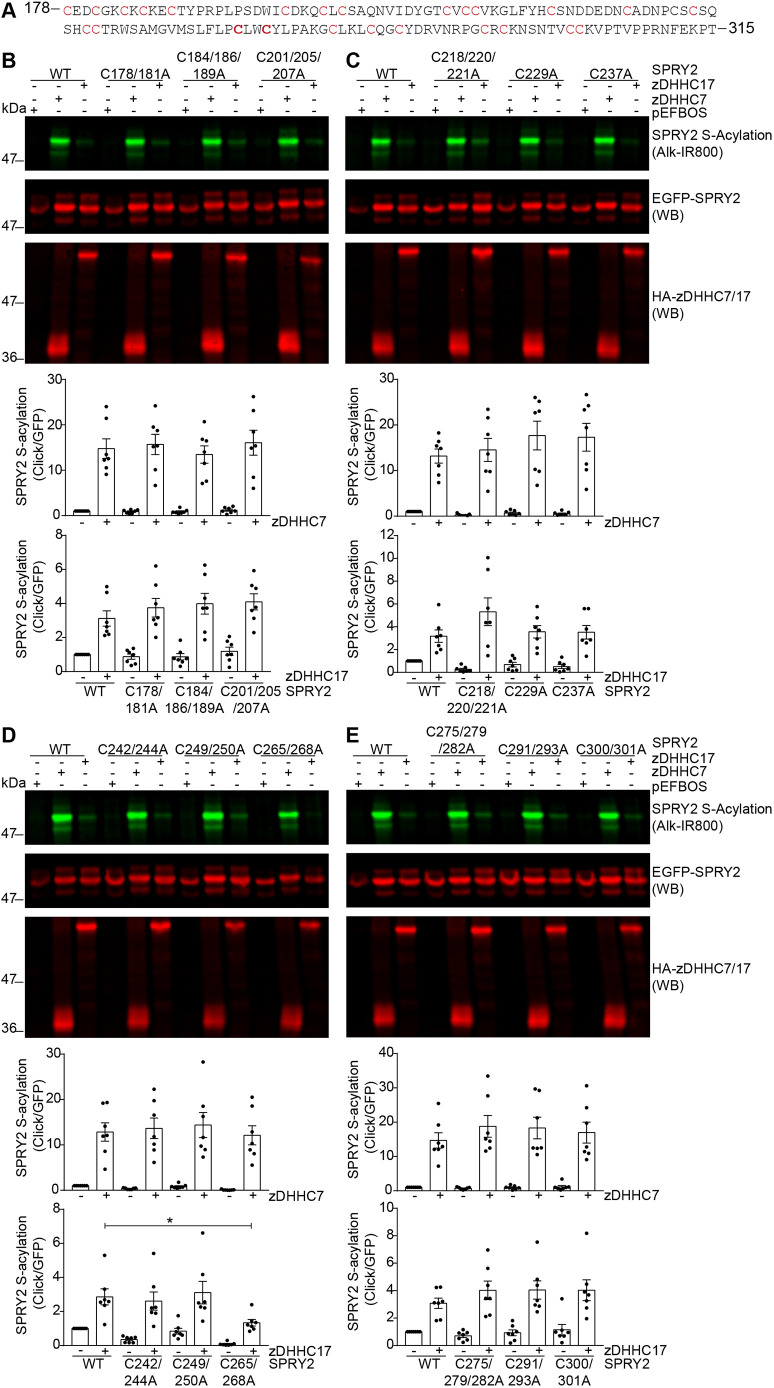
**Efficient S-acylation by zDHHC17 requires cysteine-265 and -268 within the CRD of sprouty-2.** (A) Amino acid sequence of sprouty-2 C-terminus (aa 178–315) containing the CRD (178–282) with all the cysteine residues that were mutated to alanine highlighted in red. Among these, C265 and C268 are shown in bold. (B–E) HEK293 T cells were transfected with plasmids encoding EGFP-tagged sprouty-2 WT, or one of the 12 cysteine to alanine mutants, together with pEFBOS-HA (indicated as ‘–’ in the figure), or plasmids encoding HA-tagged zDHHC17 or zDHHC7 (indicated as ‘+’ zDHHC17 or zDHHC7 in the figure). Cells were incubated with 100 μM palmitic acid azide for 4 h and labelled proteins reacted with alkyne IRdye-800 using click chemistry. S-acylation was revealed at 800 nm; GFP and HA signals were detected at 700 nm. Representative images show sprouty-2 S-acylation (top) and sprouty-2 expression levels (middle) detected on the same immunoblot, HA (bottom) was revealed for the same samples on a different immunoblot. The positions of the molecular mass markers are shown on the left. Graphs underneath images show the quantification of sprouty-2 S-acylation after normalisation. Data are pooled from seven independent experiments. Results represent mean± s.e.m.; each replicate is shown with filled circles. For clarity, only relevant statistical analysis is shown in the figure, specifically, where the S-acylation signal with zDHHC7 or zDHHC17 of a mutant protein was different to that of the WT protein with zDHHC7 or zDHHC17. In all cases, S-acylation of SPRY2 WT or sprouty-2 cysteine mutants was significantly increased by co-expressing zDHHC7 or zDHHC17, compared to the corresponding pEFBOS-HA controls. *P<0.05 (unpaired *t*-test).

The results described above suggest that zDHHC17 targets a small subset of cysteine residues in the CRD of sprouty-2, whereas zDHHC3 and zDHHC7 modify a larger number of these cysteines. To narrow down the cysteine residues targeted by zDHHC17, we performed alanine mutagenesis of all the putative S-acylation sites. All the 26 cysteine residues within the CRD of sprouty-2 (amino acids 178–301) were mutated in blocks of one, two or three residues (Fig. 2A). These cysteine mutants were then co-expressed with either zDHHC7 or zDHHC17. Fig. 2B–E shows that sprouty-2 S-acylation by zDHHC17 was significantly decreased only by mutation of cysteine-265 and -268 (referred to C265/268A in Fig. 2D) suggesting that these are preferred S-acylation sites. On the other hand, none of the sprouty-2 cysteine mutants (including the cysteine-265/268 mutant) showed reduced S-acylation when co-expressed with zDHHC7, in agreement with the more promiscuous activity of this enzyme.

**Fig. 3.**
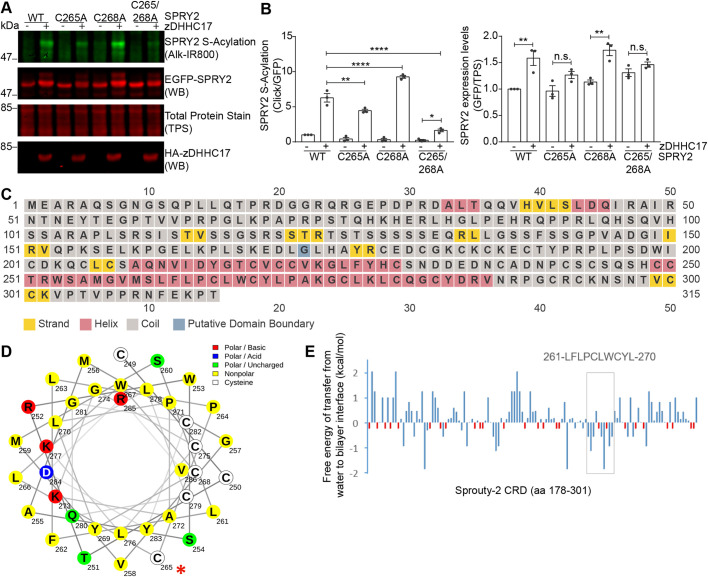
**zDHHC17 specifically S-acylates cysteine-265 within the cysteine-rich domain of sprouty-2.** HEK293 T cells were transfected with plasmids encoding EGFP-tagged sprouty-2 WT, C265A, C268A or the C265/268A double mutant, together with pEFBOS-HA (‘–’ in the figure), or plasmid encoding HA-tagged zDHHC17 (‘+’ in the figure). Cells were incubated with 100 μM palmitic acid azide for 4 h and labelled proteins reacted with alkyne IRdye-800 using click chemistry. Before performing immunoblotting, membranes were incubated with a total protein stain (TPS) and signal detected at 700 nm. S-acylation was revealed at 800 nm; GFP and HA signals were detected at 700 nm. (A) Representative image showing sprouty-2 S-acylation (top), sprouty-2 expression levels (second row) and total protein stain (TPS, third row) detected on the same immunoblot. HA (bottom) was revealed for the same samples on a different immunoblot. The positions of the molecular weight mass are shown on the left. (B) Graphs showing sprouty-2 S-acylation and expression levels after normalisation. Data shown is from one of two independent experiments. Results are mean±s.e.m.; each replicate is shown with filled circles (*n*=3). ****P<0.0001; **P<0.01; *P<0.05; n.s., not significant (one-way ANOVA). (C) The human sprouty-2 secondary structure was predicted using PSIPRED 4.0. Two main regions, encompassing amino acids 209–227 and amino acids 249–286 are predicted to form α-helices (pink shading in figure). (D) Helical wheel projection of sprouty-2 sequence 249–286, containing cysteine-265 and -268, using the NetWheels tool. Note that all the cysteine residues within this region are found on one specific side of the helix and surrounded by other hydrophobic amino acids (yellow in figure). The position of cysteine-265 is marked by an asterisk. (E) Hydrophobicity analysis of the sprouty-2 CRD. The *y*-axis shows experimentally determined free energies of transfer Δ*G* (kcal/mol) from water to a POPC interface (wif) for each residue. Cysteine residues are shown in red, and the grey box highlights amino acids 261–270 (Wimley and White, 1996).

To examine more closely the requirement for cysteine-265 and -268, these residues were also mutated individually. The results presented in Fig. 3A,B show that the S-acylation of the C265A mutant by zDHHC17 was significantly reduced compared with WT sprouty-2. In contrast, the C268A mutant did not show a loss of S-acylation. While cysteine-265 appears to be the preferential S-acylation site, there was still a substantial level of S-acylation of this mutant, and the greatest reduction was observed when both cysteine-265 and -268 were simultaneously mutated. This suggests that S-acylation of the nearby cysteine-268 may be enhanced when S-acylation of cysteine-265 is blocked.

Taken together, these results show that zDHHC17 specifically targets a small subset of cysteine residues within the CRD of sprouty-2. Structural prediction software suggests that cysteine-265 and cysteine-268 may be present in a helical stretch of the CRD (Fig. 3C). When this helical region is presented as a wheel, both these amino acids are located on a face of the helix that is largely hydrophobic (Fig. 3D). Indeed, cysteine-265 and cysteine-268 stand out from the other cysteine residues in the CRD as they are present in a hydrophobic patch (Fig. 3E). It is possible that this hydrophobic patch has an underlying membrane affinity that enhances the S-acylation potential of these two cysteine residues.

### S-acylation by both high- and low-specificity zDHHC enzymes requires specific non-cysteine residues in the CRD of sprouty-2

**Fig. 4.**
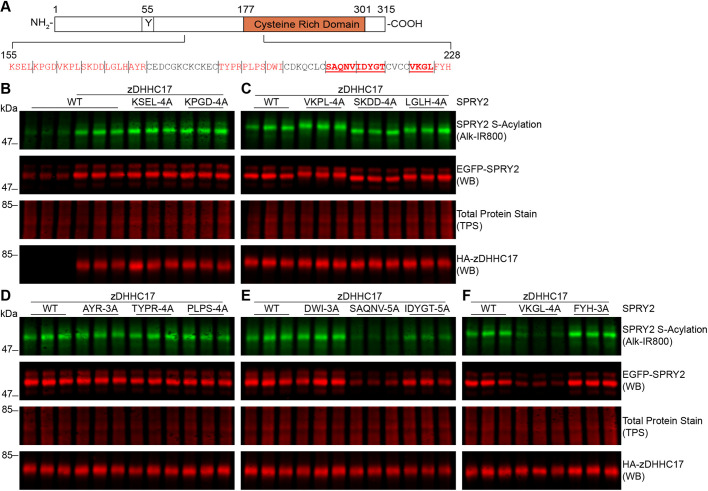
**Sprouty-2 S-acylation by zDHHC17 requires the presence of specific regions in the CRD.** (A) Schematic representation of sprouty-2 protein. The conserved Y55 is shown and the CRD is in orange. The amino acid sequence for 155–228 is shown; residues mutated in this study are shown in red, while residues that, when mutated, reduce sprouty-2 S-acylation by zDHHC17 are additionally shown in bold and underlined. (B–F) HEK293 T cells were transfected with plasmids encoding EGFP–sprouty-2 alanine mutants and HA–zDHHC17; plasmids encoding EGFP–sprouty-2 WT together with HA–zDHHC17 was used as a positive control. Cells were incubated with 100 μM palmitic acid azide for 4 h and labelled proteins reacted with alkyne IRdye-800 using click chemistry. Before performing immunoblotting, membranes were incubated with a total protein stain (TPS) and signal was detected at 700 nm. S-acylation was revealed at 800 nm; GFP and HA signals were detected at 700 nm. Each western blot shows sprouty-2 S-acylation (top), sprouty-2 expression levels (second row) and total protein stain (TPS, third row) detected on the same immunoblot, HA (bottom) was revealed for the same samples on a different immunoblot. The positions of the molecular mass markers are shown on the left.

Identification of the key features that determine the specificity of cysteine S-acylation is one of the most elusive aspects of the field (Zaballa and van der Goot, 2018). Other than the presence of a striking number of cysteine residues, no information exists on other elements that might contribute to sprouty-2 S-acylation. To investigate such specific features, we examined the amino acid region 155–229 (Fig. 4A). The reason for focusing on this region is that it is immediately downstream of the zDHHC17 recognition site (proline-154) and includes a conspicuous helical region between amino acids alanine-209 and cysteine-229 (Fig. 3C). The majority of non-cysteine residues in this region were therefore mutated to alanine in blocks of 3 to 5 amino acids, giving a total of 13 mutants. In particular, we focused on residues that occurred in stretches that lacked intervening cysteine residues (Fig. 4A). Analysis of the zDHHC17-mediated S-acylation and protein levels of each one of these mutants is shown in Fig. 4B–F. This analysis revealed that S-acylation and expression of three of these sprouty-2 mutants were reduced – SAQNV-5A (mutation of amino acids 208–212), IDYGT-5A (mutation of amino acids 213–217) and VKGL-4A (mutation of amino acids 222–225) (Fig. 4E,F). This finding suggests that there are key non-cysteine residues in the amino acid sequence 208–225, within the CRD of sprouty-2, that are required for efficient S-acylation by zDHHC17 (shown in bold red in Fig. 4A).

**Fig. 5.**
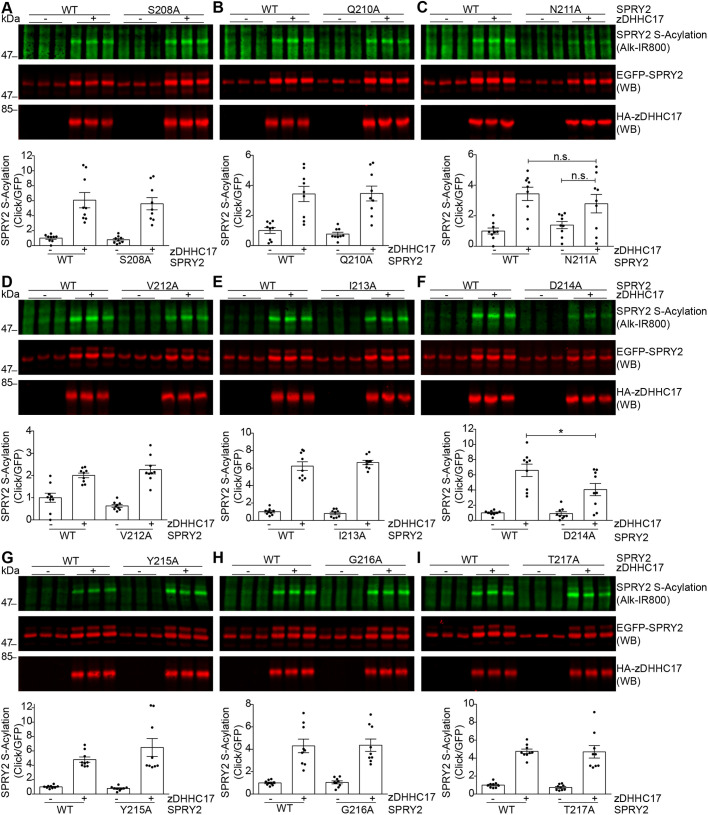
**Identification of key residues in amino acid regions 208-SAQNV-212 and 213-IDYGT-217 that are important for sprouty-2 S-acylation by zDHHC17.** Individual amino acid changes were made in the regions 208-SAQNV-212 and 213-IDYGT-217 of sprouty-2, and S-acylation and expression was evaluated for each of the resulting mutants. (A–I) HEK293 T cells were transfected with plasmids encoding EGFP-tagged sprouty-2 WT, or one of the 13 alanine mutants, together with either pEFBOS-HA (indicated as ‘–’ in figure), or HA-tagged zDHHC17 (indicated as ‘+’ in figure). Cells were incubated with 100 μM palmitic acid azide for 4 h and labelled proteins reacted with alkyne IRdye-800 using click chemistry. S-acylation was revealed at 800 nm; GFP and HA signals were detected at 700 nm. Representative images showing sprouty-2 S-acylation (top), sprouty-2 expression levels (middle) detected on the same immunoblot; HA (bottom) was revealed for the same samples on a different immunoblot. The positions of the molecular weight mass are shown on the left. Graphs underneath show sprouty-2 S-acylation after normalisation. Results are mean±s.e.m.; each replicate is shown with filled circles. Data are pooled from three independent experiments (*n*=9). *P<0.05, n.s., not significant (one-way ANOVA; for clarity, only relevant statistical differences are shown in the figure, specifically where S-acylation of mutant protein with zDHHC17 was less than that of the WT protein with zDHHC17).

**Fig. 6.**
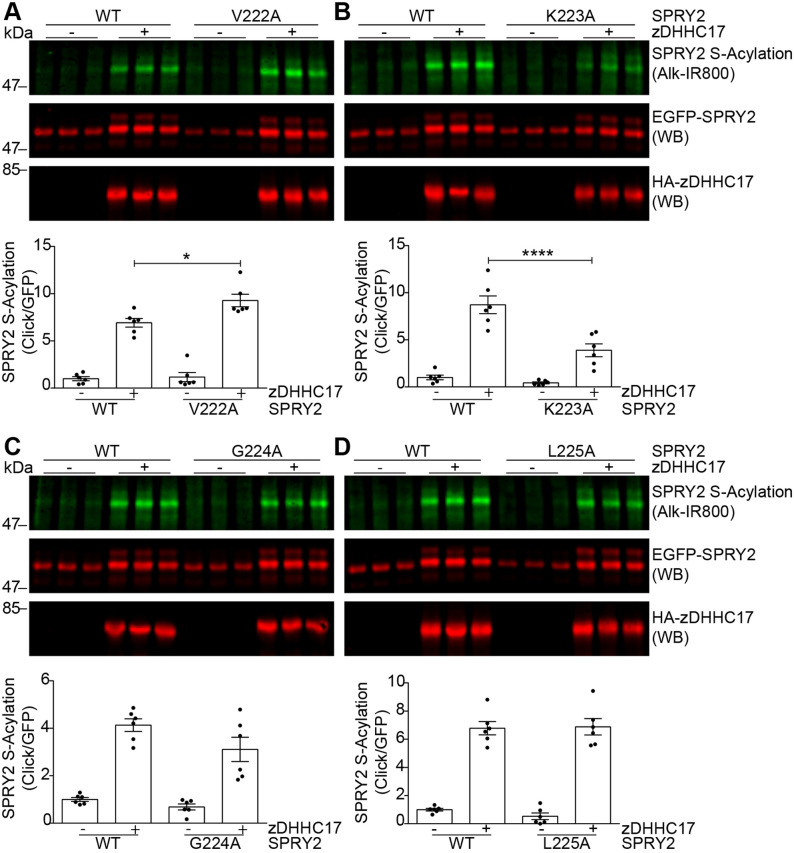
**Identification of key residues in amino acid region 222-VKGL-225 that are important for sprouty-2 S-acylation by zDHHC17.** Individual amino acid changes were made in the region 222-VKGL-225 of sprouty-2, and S-acylation and expression was evaluated for each of the resulting mutants. (A–D) HEK293 T cells were transfected with plasmids encoding EGFP-tagged sprouty-2 WT, or one of the 13 alanine mutants, together with either pEFBOS-HA (indicated as ‘–’ in figure), or HA-tagged zDHHC17 (indicated as ‘+’ in figure). Cells were incubated with 100 μM palmitic acid azide for 4 h and labelled proteins reacted with alkyne IRdye-800 using click chemistry. S-acylation was revealed at 800 nm, GFP and HA signals were detected at 700 nm. Representative images showing sprouty-2 S-acylation (top), sprouty-2 expression levels (middle) detected on the same immunoblot; HA (bottom) was revealed for the same samples on a different immunoblot. The positions of the molecular weight markers are shown on the left. Graphs underneath show sprouty-2 S-acylation after normalisation. Results are mean±s.e.m.; each replicate is shown with filled circles. Data are pooled from two independent experiments (*n*=6). ****P<0.0001, *P<0.05 (one-way ANOVA; for clarity, only relevant statistical differences are shown in the figure, specifically where S-acylation of mutant protein with zDHHC17 was less than that of the WT protein with zDHHC17).

To narrow down the residues directly involved in sprouty-2 S-acylation, we subsequently undertook additional site-directed mutagenesis where each non-cysteine amino acid within the 208–225 region was individually mutated to alanine. These mutants were then co-expressed with zDHHC17 (Figs 5 and 6). Mutation of aspartic acid-214 (D214) (Fig. 5F), and lysine-223 (K223) (Fig. 6B) resulted in a significant decrease in S-acylation by zDHHC17 compared to that seen with WT sprouty-2. Interestingly, although mutation of the amino acid region 208–212 (SAQNV) caused a loss of S-acylation (4), none of the single mutations of this region led to a significant loss in the level of S-acylation with zDHHC17 compared with WT sprouty-2 (Fig. 5A–D). This suggests that the reduced S-acylation seen with mutation of the entire region likely reflects small additive effects of multiple amino acids. In particular, and contrary to all other sprouty-2 single mutants, mutation of asparagine-211 (N211) resulted in sprouty-2 not being very susceptible to zDHHC17-mediated modification, when S-acylation for this mutant was compared in the presence or absence of zDHHC17 (Fig. 5C). Taken together, this analysis identified D214 and K223, within the CRD of sprouty-2, as key residues required for efficient S-acylation of sprouty-2 by zDHHC17, with a possible role also for N211.

**Fig. 7.**
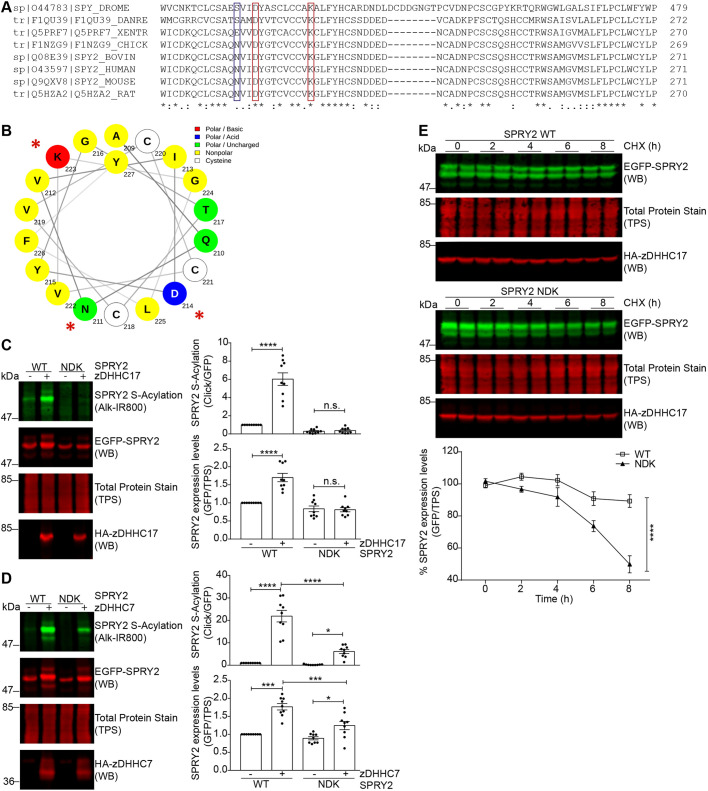
**Mutation of N211, D214 and K223 in the CRD leads to a loss of sprouty-2 S-acylation and expression mediated by both zDHHC17 and zDHHC7.** (A) CLUSTAL multiple sequence alignment of sprouty-2 protein across species. Both D214 and K223 (red rectangles in figure) are highly conserved, whereas N211 (blue rectangle in figure) is only partially conserved. Compared species: *Drosophila melanogaster* (fruit fly), *Danio rerio *(*zebrafish)*, *Xenopus tropicalis* (clawed frog), *Gallus gallus* (chicken), *Bos taurus* (cow), *Homo sapiens* (human), *Rattus norvegicus* (rat) and *Mus musculus* (mouse). (B) Helical wheel projection of sprouty-2 region encompassing amino acids 209–227, containing N211, D214 and K223, made using NetWheels software. For the secondary structure prediction of sprouty-2 refer to Fig. 4C. (C,D) HEK293 T cells were transfected with plasmids encoding EGFP-tagged sprouty-2 WT, or sprouty-2 NDK mutant together with pEFBOS-HA (indicated as ‘–’ in figure), HA-tagged zDHHC17 or zDHHC7 (indicated as ‘+’ in figure). Cells were incubated with 100 μM palmitic acid azide for 4 h and labelled proteins reacted with alkyne IRdye-800 using click chemistry. Before performing immunoblotting, membranes were incubated with a total protein stain (TPS) and signal detected at 700 nm. S-acylation was revealed at 800 nm, GFP and HA signals were detected at 700 nm. Representative images, shown on the left, are of sprouty-2 S-acylation (top), sprouty-2 expression levels (second row) and total protein stain (TPS, third row) detected on the same immunoblot. HA (bottom) was revealed for the same samples on a different immunoblot. The positions of the molecular weight mass are shown on the left. Graphs on the right show SPRY2 S-acylation and expression levels after normalisation. Results are mean±s.e.m.; each replicate is shown with filled circles. Data are pooled from three independent experiments (*n*=9). ****P<0.0001; ***P<0.001; *P<0.05; n.s., not significant (one-way ANOVA; for clarity, some statistical differences are excluded from the figure). (E) Cycloheximide-block experiments. HEK293 T cells were transfected with plasmids encoding either EGFP-tagged sprouty-2 WT or NDK mutant together with HA-tagged zDHHC17. After ∼24 h, cells were treated with 50 µg/ml cycloheximide (CHX) for 0, 2, 4, 6 or 8 h. Cells were lysed in 1× SDS sample buffer and resolved by SDS-PAGE. Before performing immunoblotting, membranes were incubated with a total protein stain (TPS) and signal detected at 700 nm. GFP and HA signals were detected at 800 and 700 nm, respectively. Representative images showing sprouty-2 expression levels (top), total protein stain (TPS, middle) and HA levels (bottom). The positions of the molecular mass markers are shown on the left. Graph underneath shows SPRY2 WT (open squares) and SPRY2 NDK (triangles) levels after normalisation at the indicated time points. Results are mean±s.e.m. Data are pooled from two independent experiments (*n*=6). ****P<0.0001 (two-way ANOVA).

 Fig. 7A shows an amino acid alignment of the region of interest from various species. Notably, both D214 and K223 are highly conserved from *Drosophila* to humans, whereas N211 is less conserved, supporting the idea of a more marginal role for this residue. In a helical wheel projection, it is interesting that the charged residues D214 and K223 are positioned on opposite sides of the helix (Fig. 7B). This evidence might suggest a broader role of these amino acids in the context of sprouty-2 S-acylation, for example, in facilitating appropriate membrane interaction rather than interaction with a specific zDHHC enzyme.

To examine whether these residues do indeed play a broader role in driving sprouty-2 S-acylation rather than allowing specific S-acylation by zDHHC17, we generated a combined mutant in which D214, K223 and N211 were replaced by alanine. This ‘NDK’ mutant was then co-expressed in HEK293 T cells with either zDHHC17 or zDHHC7. As shown in Fig. 7C,D, the combined replacement of all three amino acids in the NDK mutant with alanines, dramatically reduced both sprouty-2 S-acylation and expression levels compared to that seen for WT sprouty-2. While sprouty-2 NDK S-acylation and protein expression levels were not enhanced by zDHHC17 co-expression, zDHHC7 co-expression did have a partial effect on these parameters, but still substantially less than that observed with WT sprouty-2. Taken together, this data further support that the NDK residues, rather than facilitating S-acylation by specific zDHHC enzymes, play a more general role in sprouty-2 S-acylation.

### The NDK mutant displays more rapid degradation than WT sprouty-2

The results presented thus far clearly suggest a link between S-acylation of sprouty-2 and protein expression levels. S-acylation has previously been shown to modulate the stability of some proteins (Chamberlain and Shipston, 2015) and therefore we investigated whether this was also the case for sprouty-2. To examine this, HEK293 T cells were transfected with either WT sprouty-2 or the NDK mutant (together with zDHHC17). After overnight incubation, transfected cells were incubated in 50 µg/ml cycloheximide for 0, 2, 4, 6 or 8 h to inhibit protein synthesis and then analysed by immunoblotting. The results of this analysis showed that the NDK mutant has a significantly faster rate of degradation than the WT protein (Fig. 7E), consistent with a role for S-acylation in mediating sprouty-2 stabilisation.

### Disruption of S-acylation leads to mislocalisation of sprouty-2 and loss of plasma membrane association

**Fig. 8.**
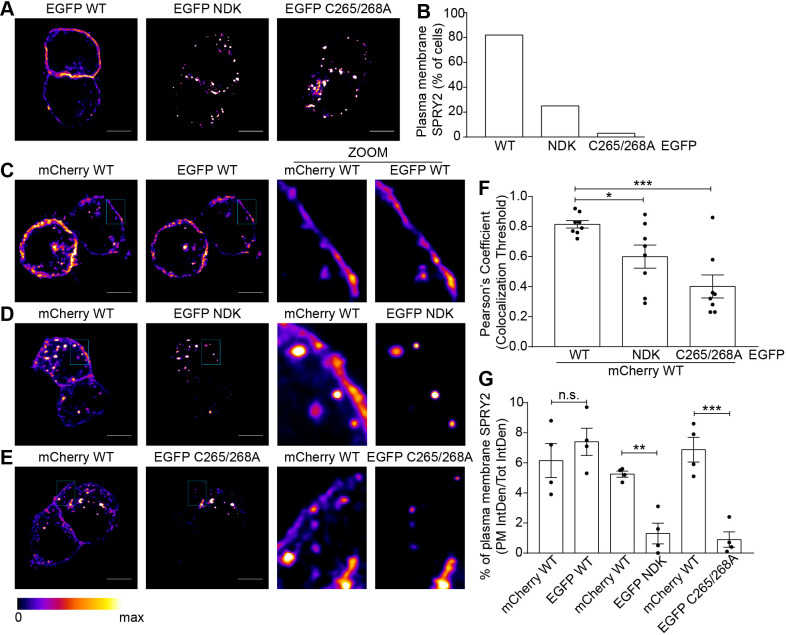
**Analysis of the localization of sprouty-2 WT and NDK and C265/268A mutants.** (A) Confocal imaging of PC12 cells transfected with plasmids encoding EGFP–sprouty-2 WT, NDK or C265/268A. Representative images are shown in figure. Scale bars: 5 μm. (B) Graph showing the percentage (%) of cells showing clear PM staining for each EGFP construct. A total of 73 cells was counted for each condition. (C–E) Confocal imaging of PC12 cells co-transfected with plasmids encoding EGFP–sprouty-2 WT, NDK or C265/268A together with plasmid encoding mCherry–sprouty-2 WT. Representative images are shown in figure for mCherry and EGFP, and a magnified view of the indicated area for both channels is shown as ‘ZOOM’. Scale bars represent 5 μm. (F) Graph showing Pearson's coefficient (*R*_tot_). Each bar shows mean±s.e.m. values of *R*_tot_; filled circles represent individual images (*n*=8). ***P<0.001, *P<0.05 (unpaired *t*-test). (G) Graph showing percentage (%) of sprouty-2 at the PM calculated as the ratio of the PM fluorescence intensity (PM IntDen) against the total fluorescence intensity (Tot IntDen) in the same cell, for both EGFP and mCherry. Each bar shows mean±s.e.m.; filled circles represent individual cells (*n*=4). ***P<0.001; **P<0.01; n.s., not significant (unpaired *t*-test).

We have shown that two distinct sprouty-2 mutants, NDK and C265/268A, have a marked inhibitory effect on sprouty-2 S-acylation. As this modification is known to regulate trafficking of other S-acylated proteins (Chamberlain and Shipston, 2015; Greaves and Chamberlain, 2007), we investigated whether these mutants have an altered distribution compared with WT sprouty-2. These experiments were performed in neuroendocrine PC12 cells, as these express transfected proteins at lower levels than HEK293 T cells. This lower expression limits cell-to-cell variability and issues with protein mistargeting. Furthermore, our previous work has shown that other substrates of zDHHC17, such as the SNARE protein SNAP25 and cysteine-string protein, are efficiently S-acylated and trafficked in PC12 cells without the need for zDHHC17 co-expression (implying that endogenous expression of this enzyme is sufficient to mediate effective S-acylation of transfected proteins) (Greaves et al., 2009b; Salaun et al., 2020). Thus, the localisation of EGFP-tagged WT sprouty-2 was compared with the EGFP-tagged C265/268A and NDK S-acylation-deficient mutants by confocal microscopy. This analysis showed that both the C265/268A and NDK mutants displayed a loss of plasma membrane localisation compared with WT sprouty-2 (Fig. 8A,B). To confirm this difference using a more quantitative methodology, PC12 cells were co-transfected with EGFP-tagged sprouty-2 WT or its NDK or C265/268A mutant, together with mCherry–sprouty-2 WT. This approach allows the localisation of the mutant and WT proteins to be compared in the same cell. As shown in Fig. 8C–E, mCherry–sprouty-2 WT colocalised with EGFP–sprouty-2 WT, whereas there was a clear visual loss of EGFP–sprouty-2 NDK and EGFP–sprouty-2 C265/268A expression at the plasma membrane compared with the WT mCherry construct. This different localisation was confirmed quantitatively by calculating the Pearson's coefficient of correlation, determined for the fluorescence intensity co-variance of the EGFP constructs with the co-expressed mCherry WT construct (Fig. 8F). To provide a more direct quantitative analysis of the observed difference in plasma membrane localisation of the mutant proteins, the fluorescence intensity of sprouty-2 at the plasma membrane was also calculated. To do this, the fluorescence intensity at the plasma membrane (PM) was expressed as a ratio against the total fluorescence intensity (PM IntDen/Tot IntDen), within the same cell and for both EGFP (WT, NDK or C265/268A) and co-expressed mCherry WT constructs. As predicted, both EGFP–sprouty-2 NDK and C265/268A mutants had a marked decrease in plasma membrane localisation compared to mCherry–sprouty-2 WT expressed in the same cells, whereas there was no significant difference in plasma membrane fluorescence of co-expressed EGFP and mCherry-tagged WT sprouty-2 proteins (Fig. 8G).

Taken together, these results clearly show that mutations that perturb sprouty-2 S-acylation lead to a loss of plasma membrane targeting of this protein.

## DISCUSSION

Sprouty proteins are major players in regulating cellular homeostasis (Kim and Bar-Sagi, 2004) and their deregulation has been linked to a number of malignancies, developmental disorders and neurological diseases (Hausott and Klimaschewski, 2019; Kawazoe and Taniguchi, 2019; Masoumi-Moghaddam et al., 2014). Sprouty-2, the focus of this study, is the most highly conserved of the sprouty proteins and its CRD was reported to undergo S-acylation almost 20 years ago (Impagnatiello et al., 2001). However, the specific zDHHC enzymes involved in sprouty-2 S-acylation and the cellular consequences of this modification are not clearly established. We previously showed that the ANK domain of zDHHC17 binds to sprouty-2 through its zDABM centred at proline-154 (Lemonidis et al., 2017b). Furthermore, zDHHC17 co-immunoprecipitated with sprouty-2 in interactome studies (Huttlin et al., 2017). Based on these data, the present study aimed to determine (1) whether sprouty-2 is a substrate of zDHHC17, (2) the specific cysteine residues within the CRD of the protein that are modified by this enzyme, (3) the influence of other (non-cysteine) residues in the CRD on sprouty-2 S-acylation, and (4) the importance of S-acylation in the context of sprouty-2 localisation and intracellular targeting. In aspects of the study, we also used the highly active and more promiscuous zDHHC3 and zDHHC7 enzymes (which colocalise at the *cis*-Golgi with zDHHC17) to provide a comparative analysis with zDHHC17. As discussed below, comparison with these high-activity enzymes supported the identification of specific cysteine residues that are targeted by zDHHC17, highlighted a more general role for specific non-cysteine residues in the S-acylation process, and showed that the restricted S-acylation pattern mediated by zDHHC17 is sufficient for full stabilisation of sprouty-2.

The key findings of this study are that, first, sprouty-2 is differentially S-acylated by zDHHC17, zDHHC7 and zDHHC3. Specifically, zDHHC17 appears to target mainly cysteine-265 and cysteine-268, whereas zDHHC3/7 mediate more expansive S-acylation of the CRD. Second, S-acylation of sprouty-2 leads to protein stabilisation. This conclusion is based on the findings that co-expression of zDHHC3, zDHHC7 or zDHHC17 enhanced the levels of sprouty-2, whereas an inactive zDHHC17 mutant did not. The similar effects of zDHHC3 and zDHHC7 versus zDHHC17 on sprouty-2 levels implies that effective stabilisation is achieved by the modification of a restricted subset of cysteine residue in the CRD (i.e. cysteine-265 and -268). Further evidence that S-acylation stabilises sprouty-2 came from the observations that the C265/268A and the NDK S-acylation-deficient mutants displayed reduced expression compared to WT sprouty-2, and indeed cycloheximide block experiments demonstrated that the S-acylation-deficient NDK mutant was degraded more quickly than the WT protein. Third, the effects of S-acylation on sprouty-2 stability/degradation do not appear to be linked to known degradation pathways of this protein as tyrosine-55 phosphorylation was not required. This suggests that S-acylation stabilises sprouty-2 by a novel mechanism. Fourth, sprouty-2 S-acylation requires specific non-cysteine residues in the CRD, including the highly conserved residues arginine-214 (D214) and lysine-223 (K223). These residues do not appear to be required for zDHHC17 interaction as their mutation also perturbed S-acylation mediated by zDHHC7, suggesting they have a more general role in the S-acylation pathway of sprouty-2 (e.g. by ensuring efficient membrane association prior to S-acylation). Finally, S-acylation regulates plasma membrane targeting of sprouty-2 in neuroendocrine PC12 cells. This conclusion is supported by the quantitative analysis of the distribution of two distinct S-acylation-deficient mutants of sprouty-2 (C265/268A and NDK mutants).

The differences in S-acylation of sprouty-2 mediated by zDHHC17 versus zDHHC3/7 were striking. This is likely to reflect the distinctly different properties of these enzymes – high activity/low specificity (zDHHC3 and zDHHC7) versus low activity/high specificity (zDHHC17). While zDHHC17 requires prior binding to substrates for successful S-acylation, zDHHC3 and zDHHC7 show a very weak or undetectable binding to their substrates (Greaves et al., 2010; Lemonidis et al., 2014, 2017a). The ANK domain of zDHHC17 recognises a specific zDABM signature in its substrate proteins, which in sprouty-2 is centred at proline-154 (Lemonidis et al., 2017b). Following this binding, the catalytic DHHC domain of zDHHC17 mediates substrate S-acylation. It is possible that interaction of the ANK domain with sprouty-2 physically limits the cysteine residues within the CRD that are accessible to the membrane-proximal DHHC catalytic domain of zDHHC17. In support of this idea, the length and flexibility of the linker region between the zDABM and the CRD of SNAP25 were recently found to be important parameters for the efficient S-acylation of SNAP25 by zDHHC17 (Salaun et al., 2020). It is also interesting to note that cysteine-265 and cysteine-268 are present in a hydrophobic patch of the CRD. Our previous work has shown that hydrophobicity of the CRD of SNAP25 and cysteine-string protein is essential for the S-acylation of these proteins (Greaves and Chamberlain, 2006; Greaves et al., 2008, 2009b). We proposed that this relates to the requirement for transient membrane association of the CRDs of these proteins for recognition and S-acylation by zDHHC17 (Greaves and Chamberlain, 2006; Greaves et al., 2008, 2009b). Thus, it is possible that the hydrophobic patch of sprouty-2 (residues 261–270 in the mouse protein) facilitates the S-acylation of cysteine-265 and cysteine-268 by mediating membrane association. A low intrinsic S-acylation activity of zDHHC17 might require cysteine residues that are targeted by this enzyme to have a longer membrane residency time in their non-acylated state. On the other hand, the high activity of zDHHC7 and zDHHC3, coupled with the lack of structural constraints imposed by a substrate recruitment domain, are two factors that may allow these highly promiscuous enzymes to modify any cysteine residues that are accessible at the membrane interface. Cysteine-265 and cysteine-268 are present in a predicted α-helical region and this helix also encompasses arginine-252 (R252), which has been previously reported to be important for PIP2 binding and correct localisation of sprouty-2 at the plasma membrane following growth factor stimulation (Lim et al., 2002). Thus, it is possible that the cysteines that are modified by zDHHC17 somehow cooperate with R252 to mediate S-acylation and/or localisation.

In addition to the cysteine residues important for sprouty-2 S-acylation by zDHHC17, we also found that aspartic acid-214 (D214) and lysine-223 (K223), within the CRD of sprouty-2 are key residues for correct S-acylation by both zDHHC17 and zDHHC7. An NDK mutant, in which these residues, together with asparagine-211 (N211) were replaced by alanine residues, completely abolished and greatly reduced sprouty-2 S-acylation by zDHHC17 and zDHHC7, respectively, suggesting that integrity in this region is required for S-acylation in general. Sequence conservation across a number of species including *Drosophila*, *Xenopus*, zebrafish, mouse and human, further supports the importance of these residues. The NDK motif is in a predicted α-helical region where the negatively charged D214 orientates on the opposite side to the positively charged K223. Given the position of these residues, it is possible that they coordinate, for example, a specific interaction between this helix and cell membranes.

Interestingly, the S-acylation of sprouty-2 was clearly linked to protein stability, and indeed S-acylation-mediated stabilisation is a recognised feature of several other proteins (Chamberlain and Shipston, 2015). It is not clear how S-acylation promotes stabilisation of sprouty-2 but this could involve changes in local structure or membrane association that mask ubiquitylation sites or degrons in the protein. Whatever the mechanism, this effect of S-acylation on expression levels is likely to be important, as sprouty-2 is downregulated, even transcriptionally silenced, in several malignancies (Kawazoe and Taniguchi, 2019). Therefore, a better understanding of the diverse mechanisms that physiologically regulate the intracellular levels of sprouty-2 is important.

One of the key roles played by S-acylation is the trafficking of soluble proteins from the Golgi to the plasma membrane (Chamberlain and Shipston, 2015; Ernst et al., 2018; Greaves et al., 2009a; Linder and Deschenes, 2007), and S-acylation of sprouty-1 and sprouty-2 was previously suggested to represent the molecular basis for their membrane association in HUVECs (Impagnatiello et al., 2001). In line with this, we found that S-acylation is a prerequisite for correct targeting of sprouty-2 to the plasma membrane. In PC12 cells, sprouty-2 WT localised both at the plasma membrane and on intracellular vesicles or puncta, whereas the C265/268A and NDK mutants displayed a marked loss of plasma membrane levels. Although the current data clearly highlights a role for S-acylation in regulating plasma membrane targeting of sprouty-2, it will be interesting in future work to investigate the interplay between dynamic S-acylation and dynamic trafficking/localisation of sprouty-2 in response to a range of different growth factors. Furthermore, although the localisation of a protein is a fundamental property linked to its cellular function, it will be important to determine how altered S-acylation impacts sprouty-2 function in relation to growth factor signalling.

This study provides the first detailed analysis of sprouty-2 S-acylation. In addition to identifying key amino acids required for S-acylation, and showing a role for this PTM in stabilisation and plasma membrane targeting of sprouty-2, the study also demonstrates differential modification of a substrate protein by different classes of zDHHC enzyme. Given the key role of sprouty-2 as a tumour suppressor, a better understanding of the physiological consequences of its S-acylation will provide new insight that may have therapeutic relevance.

## MATERIALS AND METHODS

### Antibodies

Mouse anti-GFP antibody (Clontech, JL8, 1:3000) was purchased from Takara (Saint-Germain-en-Laye, France). Rat anti-HA antibody (Roche, 3F10, 1:1000) was from Sigma (Poole, UK). REVERT Total Protein Stain Kit and secondary antibodies conjugated to IRdye (used at 1:15,000 dilution) were purchased from Li-COR Biosciences (Cambridge, UK) and used accordingly to the manufacturer's protocol.

### Plasmids

Mouse zDHHC17, zDHHC7 and zDHHC3 were subcloned in pEF-BOS HA and kindly provided by Prof. Masaki Fukata (National Institute of Physiological Sciences, Okazaki, Japan; Fukata et al., 2004), whereas the DHHA17 mutant was generated by site-directed mutagenesis. The cDNA encoding mouse SPRY2 WT was cloned by Gateway Technology into pEGFP-C2 (Lemonidis et al., 2014) or pmCherry-C1, according to manufacturer's guidelines (Life Technologies, Inc.). All the sprouty-2 mutants described were generated by site-directed mutagenesis. The oligonucleotide primers were designed using QuikChange Primer Design software (Agilent, Santa Clara, CA) and purchased from Sigma (Poole, UK). All plasmids were sequenced by Eurofins Genomics (GATC, Constance, DE).

### Cell culture and transfection

HEK293 T cells (ATCC, CRL-3216) were maintained in DMEM GlutaMAX (Thermo Fisher Scientific) supplemented with 10% fetal bovine serum (FBS). PC12 cells (ATCC, CRL-1721) were maintained in Advanced RPMI 1640 medium (Thermo Fisher Scientific) supplemented with 10% horse serum (HS), 5% FBS and 1% glutamine. All cells were grown at 37°C in a humidified atmosphere containing 5% CO_2_.

For click chemistry assays and cycloheximide-block experiments, HEK293 T were seeded on 24-well poly-D-lysine-coated plates (Corning BioCoat, VWR, UK) and transfected with 0.66 μg of pEF-BOS HA plasmid (either empty plasmid or with cDNA for zDHHC enzymes) and 0.33 μg of EGFP–sprouty-2 (either WT or mutants). Polyethylenimine (PEI) 1 mg/ml stock (Linear PEI, MW 25000, #43896, Alpha Aesar, UK) was used at a ratio PEI:DNA of 2:1 to mediate cell transfection. The DNA/PEI mix was incubated at room temperature for 20 min before being added to the cells.

For confocal imaging, PC12 cells were seeded on 12 mm BD poly-D-lysine-coated coverslips (Thermo Fisher Scientific, UK). 0.2 μg of EGFP-tagged sprouty-2 WT, NDK or C265/268A plasmids were either transfected alone or in combination with 0.2 μg of mCherry–sprouty-2 WT using Lipofectamine 2000 (Invitrogen, UK) at a ratio of 2 μl of Lipofectamine 2000 to 1 μg plasmid. The DNA/Lipofectamine mix was incubated for 20 min at room temperature and then added to the cells.

### Click chemistry

At ∼24 h post-transfection, HEK293 T cells were washed once with PBS and then incubated in 100 μM of C16:0 Azide (Greaves et al., 2017) in 500 μl of serum-free DMEM supplemented with 1 mg/ml fatty-acid free BSA (Sigma, UK) and incubated at 37°C. After 4 h, cells were washed once in PBS and lysed in 100 μl of lysis buffer containing 50 mM Tris-HCl pH 8, 0.5% SDS and protease inhibitor cocktail (P8340, Sigma, UK). To each 100 μl of cell lysate, an equal volume (100 μl) of freshly made click reaction mix containing 5 μM of Alkyne-IRDye 800CW (929-60002, Li-COR, UK), 4 mM CuSO4 (451657, Sigma, UK), 400 μM Tris[(1-benzyl-1H-1,2,3-triazol-4-yl)methyl]amine (678937, Sigma, UK) and 8 mM ascorbic acid (A15613, Alpha Aesar, UK), was added. The click reaction was carried out at room temperature with end-over-end rotation for 1 h. Samples were then supplemented with 67 μl of 4× SDS sample buffer containing 100 mM DTT and heated for 5 min at 95°C. Samples were then resolved by SDS-PAGE and transferred to nitrocellulose membranes. Before performing immunoblotting, membranes were incubated with a total protein stain. Click, total protein stain and antibody signals were visualised using a Li-COR Odyssey infrared scanner and quantified with ImageStudio software (Li-COR, USA). Sprouty-2 S-acylation was quantified as a ratio of the click signal against the corresponding protein signal (EGFP immunoblot). Sprouty-2 expression levels were quantified as a ratio of protein signal (EGFP immunoblot) against the corresponding total protein stain (TPS).

### Cycloheximide treatments

At ∼24 h post-transfection, cells were incubated with 50 µg/ml cycloheximide (CHX; C-7698, Sigma, UK). After 0, 2, 4, 6 or 8 h treatments, cells were washed once with PBS, lysed in 1× SDS sample buffer containing 25 mM DTT and heated for 5 min at 95°C. Samples were processed for immunoblotting as described above. Sprouty-2 levels were quantified as a ratio of protein signal (EGFP immunoblot) against the corresponding TPS.

### Confocal imaging and analysis

PC12 cells were prepared for immunofluorescence analysis at ∼48 h post-transfection. Cells were washed twice in PBS and fixed in 500 μl of 4% formaldehyde for 30 min at room temperature. After an initial wash in PBS and a second one in dH_2_O, coverslips were air-dried, and mounted on glass slides in Mowiol. All images were acquired as *z*-stacks using a Leica SP8 confocal microscope in Lightning mode. Image analysis was performed using ImageJ-Fiji software and data was analysed in GraphPad 7.0.

To calculate the Pearson's coefficient (*R*_tot_), the colocalisation threshold function was used to analyse eight different images.

To compare the sprouty-2 localisation at the plasma membrane between mCherry–sprouty-2 WT and EGFP–sprouty-2 WT or mutant constructs, a single slice was chosen for each image. The fluorescence intensity associated with the plasma membrane (PM IntDen) and the whole cell (Tot IntDen) was calculated for identical regions of interest for both the mCherry (WT) and EGFP (WT or mutant) signal. The amount of sprouty-2 at the plasma membrane was derived as a ratio of the fluorescence intensity at the PM against the fluorescence intensity of the whole cell (PM IntDen/Tot IntDen).

### Secondary structure prediction, helical wheel projections and sprouty-2 sequence alignment

To predict the secondary structure of sprouty-2, we employed PSIPRED 4.0 Protein Analysis Workbench (from the UCL Department of Computer Science; Buchan and Jones, 2019; Jones, 1999). This tool takes into account outputs from different platforms (i.e. PSI-BLAST) providing a prediction accuracy of 84.2% (Buchan and Jones, 2019). Briefly, the amino acid sequence of human sprouty-2 was obtained from UniProtKB in FASTA format and uploaded on PSIPRED 4.0, which returned the predicted secondary structure.

NetWheels software was used to generate helical wheel projections. Briefly, the sequences of interest were used as inputs to draw helical wheel projections where each amino acid is shown on the perimeter of a circle (representing the helix) and the angle of rotation of every three residues is fixed to 100° (Mól et al., 2018, preprint).

A number of sprouty-2 sequences from different species were aligned using the Clustal Omega program (EMBL-EBI). Briefly, input sequences in FASTA format were obtained from UniProtKB and directly uploaded on the program.

### Hydrophobicity analysis

Hydrophobicity analysis of the sprouty-2 CRD (amino acids 178–301) was performed using experimentally determined pentapeptide free energies of transfer Δ*G* (kcal/mol) from palmitoyloleoylphosphatidylcholine (POPC) bilayer to water, for each amino acid (Wimley and White, 1996). The degree of hydrophobicity/hydrophilicity for each residue (Δ*G* values from water to POPC interface; *y* axis) was plotted against each residue of the analysed sequence (*x* axis) using Excel software.

### Statistical analysis

All data analysis was performed using GraphPad Prism 7.0 (San Diego, CA, USA). Differences were analysed either with one-way ANOVA followed by Tukey's multiple comparison, two-way ANOVA, or unpaired Student *t*-test, as indicated in figure legends. In all graphs, the mean±s.e.m. is plotted, and the number of replicates is indicated in both the figures and their legends. For significant results, ****P<0.0001, ***P<0.001, **P<0.01 and *P<0.05.

## Supplementary Material

Supplementary information

Reviewer comments
