# Supplementary material for: Identification of key features required for efficient S-acylation and plasma membrane targeting of sprouty-2
Source: J Cell Sci. 2020 Nov 5;133(21):jcs249664. doi: 10.1242/jcs.249664 (PMC7657471; doi:10.1242/jcs.249664)
Supplement: Supplementary information [file joces-133-249664-s1.pdf]

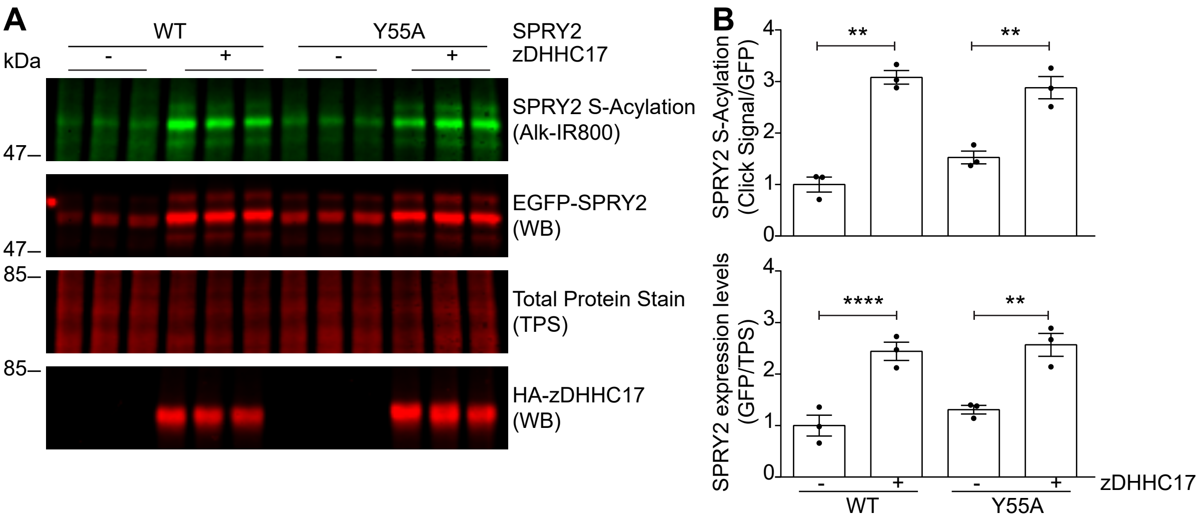

Figure S1. S-acylation by zDHHC17 promotes Sprouty-2 stabilisation independently from phosphorylation at the highly conserved tyrosine-55.

a) HEK293T cells were transfected with plasmids encoding EGFP-tagged SPRY2 WT or Y55A, together with pEFBOS-HA (indicated as "-" in figure), or plasmid encoding HA-tagged zDHHC17 WT (indicated as "+" in figure). Cells were incubated with 100 µM palmitic acid azide for 4 h and labelled proteins reacted with alkyne IRdye-800 using click chemistry. Before performing immunoblotting, membranes were incubated with a total protein stain (TPS) and the signal was detected at 700 nm. S-acylation was revealed at 800 nm, GFP and HA signals were detected at 700 nm. **a)** Representative image showing SPRY2 S-acylation (*Top*), SPRY2 expression levels (*Middle top*) and total protein stain (TPS, *Middle bottom*) detected on the same immunoblot. HA (*Bottom*) was revealed for the same samples on a different immunoblot. The positions of the molecular weight markers are shown on the left. **b)** Graphs showing SPRY2 S-acylation and expression levels after normalisation. Data shown is from one of two independent experiments. Each bar shows mean values ± s.e.m; filled circles represent individual cells. Results were analysed by one-way ANOVA. Samples co-expressing zDHHC17 were compared with the corresponding samples without zDHHC17 (**** denotes P <0.0001, ** P <0.01, n=3).

Journal of Cell Science • Supplementary information
