# Supplementary material for: Identification of key features required for efficient S-acylation and plasma membrane targeting of sprouty-2
Source: J Cell Sci. 2020 Nov 5;133(21):jcs249664. doi: 10.1242/jcs.249664 (PMC7657471; doi:10.1242/jcs.249664)
Supplement: Reviewer comments [file joces-133-249664_review_history.pdf]

Journal of Cell Science

Identification of key features required for efficient S-acylation and plasma membrane targeting of sprouty-2

Carolina Locatelli, Kimon Lemonidis, Christine Salaun, Nicholas C.O. Tomkinson and Luke H. Chamberlain
DOI: 10.1242/jcs.249664

Editor: John Heath

Review timeline
Original submission: 29 May 2020
Editorial decision: 6 July 2020
First revision received: 4 September 2020
Accepted: 30 September 2020

Original submission

First decision letter

MS ID#: JOCES/2020/249664

MS TITLE: Identification of key features required for efficient S-acylation and plasma membrane targeting of Sprouty-2

AUTHORS: Carolina Locatelli, Kimon Lemonidis, Christine Salaun, Nicholas C.O. Tomkinson, and Luke H Chamberlain
ARTICLE TYPE: Research Article

We have now reached a decision on the above manuscript.

To see the reviewers' reports and a copy of this decision letter, please go to: https://submit-jcs.biologists.org and click on the 'Manuscripts with Decisions' queue in the Author Area. (Corresponding author only has access to reviews.)

As you will see, the reviewers raise a number of substantial criticisms that prevent me from accepting the paper at this stage. They suggest, however, that a revised version might prove acceptable, if you can address their concerns. In particular, referee 1 thinks there are inconsistencies between the data and your interpretation.

If you think that you can deal satisfactorily with the criticisms on revision, I would be pleased to see a revised manuscript. We would then return it to the reviewers.

We are aware that you may currently be unable to access the lab to undertake experimental revisions. If it would be helpful, we encourage you to contact us to discuss your revision in greater detail. Please send us a point-by-point response indicating where you are able to address concerns raised (either experimentally or by changes to the text) and where you will not be able to do so within the normal timeframe of a revision. We will then provide further guidance. Please also note that we are happy to extend revision timeframes as necessary.

Please ensure that you clearly highlight all changes made in the revised manuscript. Please avoid using 'Tracked changes' in Word files as these are lost in PDF conversion.

© 2020. Published by The Company of Biologists under the terms of the Creative Commons Attribution License (https://creativecommons.org/licenses/by/4.0/).

I should be grateful if you would also provide a point-by-point response detailing how you have dealt with the points raised by the reviewers in the 'Response to Reviewers' box. Please attend to all of the reviewers' comments. If you do not agree with any of their criticisms or suggestions please explain clearly why this is so.

Reviewer 1

Advance summary and potential significance to field

In this manuscript, the authors co-express an eGFP-Spry2 fusion protein and different S-acetyltransferase enzymes (namely zDHHC 17, 7 and 3 and showed that the expression of the acetyltransferase enzymes causes an acetylation of Spry2 which was not visible when the cotransfected enzyme was inactive. This acetylation was more pronounced when zDHHC7 and zDHHC3 were introduced as compared to the expression of zDHHC. In all cases the expression of Spry2 was augmented, although the level of acetylation did not correlate with the expression levels. Furthermore the authors performed an extensive mutation analysis to identify the aa necessary for the acetylation of Spry2. For zDHHC 17, they found that the lack of 2 cysteins 265 and 268 abolished the acetylation. In this case also the elevated expression of Spry2 levels were not observed. Although this is true for the presented blot, there are in addition some mutations where acetylation seems uninfluenced although the Spory2 levels are not increased upon acetylation. Further analysis revealed that D214 and K223 were important for the acetylation and stabilization of Spry2 byzDHHc17. A triple mutated Spry2 (N211A/D214A and K223A) is no more acetylated and stabilized by zDHHC 17. In the presence of zDHHC7 both acetylation and stabilization of Spry2 can be still observed in this triple mutant, but the extent of the reactions is weakened as compared to the wildtype Spry2 protein. Finally, the authors express the Spry2 mutants in PC19 cells and show that the NDK and C265/268 mutants of Spry2 fail are less associated to the plasma membrane than the wt Spry2 protein. In this cell line (as documented by citing papers) acetylation of other proteins can be observed, if endogenous zDHHC 17 indeed can acetylate Spry2 and thereby influence its localization is not shown.

Comments for the author

In summary, the presented manuscript shows that zDHHC enzymes are able to acetylate Spry2 protein when proteins are overexpressed and identified regions within the Spry2 protein which contribute to the efficient acetylation of the protein by at least zDHHC17. Further functional impact of these observations are missing. The conclusions that Spry2 half-life and localization (and under which circumstances) is dependent on this acetylation are not sufficiently substantiated by the provided data.

Reviewer 2

Advance summary and potential significance to field

The paper reports a detailed examination of the requirements for palmitoylation in the Sprouty 2 protein by identifying the sites and the factors that control acylation as well as identifying probable enzymes. This is a significant contribution to the field because it extends our limited knowledge of this post translational modification and its importance in cellular function.

Comments for the author

As indicated above I believe the manuscript presents important new findings that are significant for the field. The data is presented in a straightforward manner with clear descriptions of the methods used.

If there is anything that needs to be added it would be a discussion of why they limited the study to only the 3 DHHC's.

The manuscript should be accepted.

© 2020. Published by The Company of Biologists under the terms of the Creative Commons Attribution License (https://creativecommons.org/licenses/by/4.0/).

First revision

Author response to reviewers' comments

Reviewer 1
We thank the reviewer for reading our manuscript and for highlighting issues for our attention. We believe that the manuscript is significantly improved as a result of the changes that we made in response to the reviewer's critique.
The main issue raised by reviewer #1 is that they felt that "conclusions that Spry2 half-life and localization (and under which circumstances) is dependent on S-acylation are not sufficiently substantiated by the provided data"
The conclusion that S-acylation affects plasma membrane targeting of Sprouty-2 was based on the localisation of two distinct S-acylation-deficient mutants. Our approach for identifying the loss of plasma membrane targeting of these mutants involved careful quantification (using two different methods) of the localisation of the S-acylation mutants compared with wild-type Sprouty-2 expressed in the same cell. This approach limits any influence of cell variability on the results obtained. Although we believe that the quantitative approach used in these experiments is rigorous, we were concerned that the reviewer was not convinced about the different localisation of the mutant constructs. Therefore, we have made the following changes and additions: (i) we have presented the representative images in a different format, which includes zoomed images that clearly highlight the lack of plasma membrane staining for the S-acylation mutants; and (ii) we have also added new images and quantification from singly transfected cells to rule out any effect of interference between EGFP-tagged mutant proteins and mCherry-tagged wild-type Spry2 in the original experiment. This new data, presented in Figure 8A-B, confirms the loss of plasma membrane signal for the S-acylation-deficient mutants.
The reviewer's comment also included a query about "under what circumstances" these effects of S-acylation occur. This is clearly an important issue that will require extensive analysis and is something that we are looking to explore more thoroughly in future work. For the current study, we have highlighted this in the Discussion section as an important issue to address in future work. We hope that this is acceptable. The added text reads: "Although the current data clearly highlights a role for S-acylation in regulating plasma membrane targeting of Sprouty-2, it will be interesting in future work to investigate the interplay between dynamic S-acylation and dynamic trafficking/localisation of Sprouty2 in response to a range of different growth factors. Furthermore, although the localisation of a protein is a fundamental property linked to its cellular function, it will be important to determine how altered S-acylation impacts Sprouty2 function in relation to growth factor signalling." (lines 416-421 of revised manuscript).
The other issue raised was that the reviewer felt we had not convincingly demonstrated a link between S-acylation and Sprouty-2 half-life. To address this concern, we analysed one of the S-acylation-deficient mutants (the NDK mutant) in cycloheximide-block experiments. Here, cells were transfected with wild-type Sprouty-2 or the NDK mutant (in the presence of zDHHC17). After overnight incubation, cycloheximide was added to the cells to block protein translation, allowing analysis of the loss of Sprouty-2 immunoreactivity as a measure of protein stability. We found that the NDK mutant was indeed degraded significantly faster than the wild-type protein, supporting the conclusion that S-acylation affects Sprouty-2 half-life. This new data is presented as Figure 7E (and described in lines 272-281) in the revised manuscript and we believe this is an important addition and appreciate the reviewer's suggestion.

Reviewer 2
We thank the reviewer for reading our manuscript and for the helpful suggestions. The reviewer noted that the "new findings are significant for the field" and suggested the manuscript should be accepted. They asked that we include some discussion of why the study was limited to three DHHC's.
The central focus of this study was on zDHHC17 (as it is a known interactor of Sprouty-2) and the inclusion of zDHHC3/7 was really to provide a comparative analysis with zDHHC17- we chose these enzymes as they are known to be highly active and promiscuous. We have added/modified text in the Discussion section to try and better explain this. The following text has been added to the Discussion section and it describes the central conclusions of the study, the rationale for including analyses of zDHHC3/7, and description of where the analysis of zDHHC3/7 alongside zDHHC17 has enhanced our understanding of Sprouty-2 S-acylation (Discussion, lines 329-360):

© 2020. Published by The Company of Biologists under the terms of the Creative Commons Attribution License (https://creativecommons.org/licenses/by/4.0/).

“[…]Based on these data, the present study aimed to determine: (i) if Sprouty-2 is a substrate of zDHHC17, (ii) the specific cysteines within the CRD of the protein that are modified by this enzyme, (iii) the influence of other (non-cysteine) residues in the CRD on Sprouty-2 S-acylation, and (iv) the importance of S-acylation in the context of Sprouty-2 localisation and intracellular targeting. In aspects of the study, we also used the highly active and more promiscuous zDHHC3 and zDHHC7 enzymes (which co-localise at the cis Golgi with zDHHC17) to provide a comparative analysis with zDHHC17. As discussed below, comparison with these high activity enzymes supported the identification of specific cysteines that are targeted by zDHHC17, highlighted a more general role for specific non-cysteine residues in the S-acylation process, and showed that the restricted S-acylation pattern mediated by zDHHC17 is sufficient for full stabilisation of Sprouty-2.
The key findings of this study are that (i) Sprouty-2 is differentially S-acylated by zDHHC17, zDHHC7, and zDHHC3. Specifically, zDHHC17 appears to target mainly cysteine-265 and cysteine-268, whereas zDHHC3/7 mediate more expansive S-acylation of the CRD; (ii) S-acylation of Sprouty-2 leads to protein stabilisation. This conclusion is based on the findings that zDHHC3/7/17 co-expression enhanced the levels of Sprouty-2, whereas an inactive zDHHC17 mutant did not. The similar effects of zDHHC3/7 versus zDHHC17 on Sprouty-2 levels implies that effective stabilisation is achieved by the modification of a restricted subset of cysteines in the CRD (i.e. cysteine-265/268). Further evidence that S-acylation stabilises Sprouty-2 came from the observations that the C265/268A and NDK S-acylation-deficient mutants displayed reduced expression compared to wild-type Sprouty-2, and indeed cycloheximide-block experiments demonstrated that the S-acylation-deficient NDK mutant was degraded more quickly than the wild-type protein; (iii) The effects of S-acylation on Sprouty-2 stability/degradation do not appear to be linked to known degradation pathways of this protein as tyrosine-55 phosphorylation was not required. This suggests that S-acylation stabilises Sprouty-2 by a novel mechanism. (iv) Sprouty-2 S-acylation requires specific non-cysteine residues in the CRD, including the highly conserved residues arginine-214 (D-214) and lysine-223 (K-223). These residues do not appear to be required for zDHHC17 interaction as their mutation also perturbed S-acylation mediated by zDHHC7, suggesting they have a more general role in the S-acylation pathway of Sprouty-2 (e.g. by ensuring efficient membrane association prior to S-acylation); and (v) S-acylation regulates plasma membrane targeting of Sprouty-2 in neuroendocrine PC12 cells. This conclusion is supported by the quantitative analysis of the distribution of two distinct S-acylation-deficient mutants of Sprouty-2 (C265/268A and NDK mutants).”

Second decision letter

MS ID#: JOCES/2020/249664

MS TITLE: Identification of key features required for efficient S-acylation and plasma membrane targeting of Sprouty-2

AUTHORS: Carolina Locatelli, Kimon Lemonidis, Christine Salaun, Nicholas C.O. Tomkinson, and Luke H Chamberlain
ARTICLE TYPE: Research Article

I am happy to tell you that your manuscript has been accepted for publication in Journal of Cell Science, pending standard ethics checks.

Reviewer 1

Advance summary and potential significance to field

In the revised manuscript, the author have improved the discussion. Additionally they provide new data allowing the conclusions that acetylation can influence Spry2 protein stability and that plasma membrane targeting of Spry2 is influenced by the sequences necessary for acetylation. Although investigations on the mechanisms regulating the observed acetylation is still missing. The

© 2020. Published by The Company of Biologists under the terms of the Creative Commons Attribution License (https://creativecommons.org/licenses/by/4.0/).

manuscript in its new form provides sufficant information to justify its publication. Therefore I recommend to publish the manuscript in the revised form.

Comments for the author

In the revised manuscript, the author have improved the discussion. Additionally they provide new data allowing the conclusions that acetylation can influence Spry2 protein stability and that plasma membrane targeting of Spry2 is influenced by the sequences necessary for acetylation. Although investigations on the mechanisms regulating the observed acetylation is still missing. The manuscript in its new form provides sufficant information to justify its publication. Therefore I recommend to publish the manuscript in the revised form.

Reviewer 2

Advance summary and potential significance to field

The prior assessment of significance is the same:

The paper reports a detailed examination of the requirements for palmitoylation in the Sprouty 2 protein by identifying the sites and the factors that control acylation as well as identifying probable enzymes. This is a significant contribution to the field because it extends our limited knowledge of this post translational modification and its importance in cellular function.

Comments for the author

I think the additions made the manuscript stronger.

The manuscript should be accepted.

© 2020. Published by The Company of Biologists under the terms of the Creative Commons Attribution License (https://creativecommons.org/licenses/by/4.0/).
